# The Burden of Managing Medicines for Older People With Sensory Impairment: An Ethnographic-Informed Study

**DOI:** 10.1177/23337214241253410

**Published:** 2024-05-16

**Authors:** Peter Fuzesi, Kirsten Broadfoot, Marilyn Lennon, Sabrina Anne Jacob, Leah Macaden, Annetta Smith, Tomas Welsh, Margaret C. Watson

**Affiliations:** 1 University of Strathclyde, Glasgow, Scotland, UK; 2 Sterena Consultancy, Cromarty, Scotland, UK; 3 Strathclyde Institute of Pharmacy and Biomedical Sciences, Glasgow, Scotland, UK; 4 The University of Edinburgh, UK; 5 University of the Highlands and Islands, Inverness, Scotland, UK; 6 Research Institute for the Care of Older People, Bath, UK; 7 Royal United Hospitals Bath, UK; 8 University of Bristol, UK

**Keywords:** medication self-management, aged, vision disorders, hearing loss, qualitative research

## Abstract

**Background:** Older age is associated with increased prevalence of sensory impairment and use of medicines. **Objectives:** To explore the daily “medicine journey” of older people with sensory impairment. **Methods:** The study used ethnographic-informed methods (using audio-, photo- and video-recordings, diary notes and semi-structured interviews with researchers) and involved community-dwelling adults (aged > 65) in Scotland, with visual and/or hearing impairment and using >4 medicines. Data analysis used the constant comparative method. **Results:** Fourteen older people with sensory impairment participated and used a mean of 11.0 (SD 5.0) medicines (range 5–22). Participants reported difficulties with medicine ordering, obtaining, storage, administration and disposal. They used elaborate strategies to manage their medicines including bespoke storage systems, fixed routines, simple aids, communication, and assistive technologies. **Conclusion:** Older people with sensory impairment experience substantial burden, challenges and risk with medicines management. Tailored medicine regimens and assistive technologies could provide greater support to older people with sensory impairment.

## Introduction

Life expectancy is increasing in every geographical region around the world (United Nations, 2022). Whilst this reflects improved living conditions for millions of people, longevity is typically accompanied by increased prevalence of long-term health conditions including visual and/or hearing impairment (hereafter referred to as sensory impairment)(Barnett et al., 2012; World Health Organisation, 2019c, 2022). Sensory impairment can impact older people’s independence (Hajek & König, 2020) and wellbeing (Crews & Campbell, 2004; Pan et al., 2016). Compared with people of the same age, those with sensory impairment are more likely to experience falls and report higher rates of co-morbid conditions including heart disease and stroke (Crews & Campbell, 2004).

Medicines are the most frequently used healthcare intervention, particularly for long-term conditions. In the United Kingdom (UK), the concept of medicines optimization was introduced to encourage prescribers and other healthcare professionals to help patients “*make the most of their medicines*” by working in partnership to adopt a person-centered approach to their healthcare and their inclusion in the decision-making process (Royal Pharmaceutical Society, 2013). Medicines optimization comprises four principles: Principle 1, the patient’s experience; Principle 2, evidence-based choice of medicines; Principle 3, maximizing the safe use of medicines; and Principle 4, adopting medicine optimization into routine practice (Royal Pharmaceutical Society, 2013). The safe and effective use of medicines can sometimes be enhanced with the use of assistive technologies that is, “products or systems that support and help individuals. . . to improve or maintain their daily quality of life by easing or compensating for an injury or disability” (MHRA, 2021). Whilst a growing range of medicine-related assistive technologies is available there is a paucity of evidence of their effectiveness in relation to their use by people with sensory impairment (Cooper et al., 2023).

**Figure 1.**
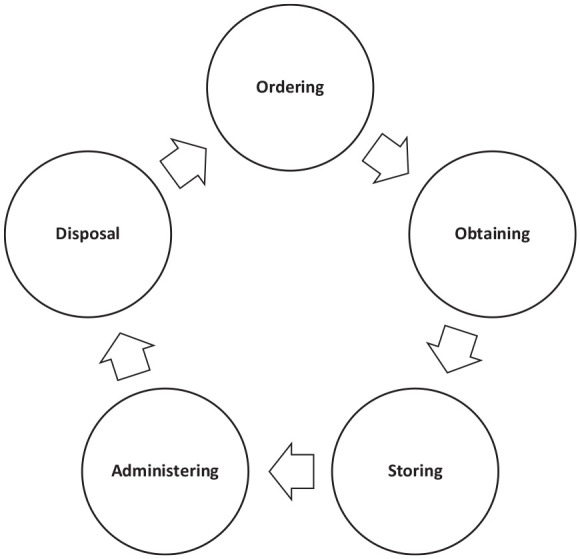
Stages of the medicine journey.

Despite the magnitude of aging populations and their inherent multi-morbidities and polypharmacy (Masnoon et al., 2017; Page et al., 2019; Petchey & Gentry, 2019; Rochon et al., 2021), there has been minimal exploration of the medicine-related needs of older people with sensory impairment (Killick et al., 2018). Our earlier research used the concept of the medicine journey comprising five stages (ordering, obtaining, storing, administering, disposal) (Figure 1) and provided novel but incomplete insight into the experience of older people with sensory impairment and their medicines (Alhusein et al., 2018; Smith et al., 2019). This included only limited exploration of the use of assistive technologies to support the medicine journey. As such, the purpose of this current study was to undertake a more detailed exploration of the patient experience (Principle 1, Medicines Optimization) (Royal Pharmaceutical Society, 2013) throughout each stage of their medicine journey, including any assistive technologies and strategies used, with the intention of using this data to inform the future development of services and products to facilitate safe medicine journeys.

## Methods

This study used ethnographic-informed methods including indirect observation using a combination of home-based photo-video and audio-diaries, and semi-structured interviews using Zoom or by telephone. Due to the Covid-19 pandemic, the original ethnographic study that had been planned could not be undertaken because the researchers were not permitted to undertake direct observation of the participants in their homes (Leverton et al., 2019), communities or workplaces. As such, the methods were *ethnographic-informed* because whilst these data *were* gathered “in naturally occurring settings” (typically the participants’ homes) it was the participants themselves who undertook the majority of data collection about their “ordinary activities and social meanings” associated with their medicines (Brewer, 2000, p. 10).

### Sampling and Recruitment

Participants were eligible for inclusion if they were community-dwelling adults ≥65 years, had hearing and/or visual impairment, used ≥4 medicines (prescription and non-prescription, on a regular basis), and lived in Scotland. No definition of hearing and/or visual impairment was used (participants self-reported their sensory impairment) and sensory function was not assessed. The intended sample size was 15 that is, five each with hearing, visual and dual impairment. Purposive sample recruitment used social media (Twitter, now X), professional networks, and third-sector organizations for example, Health and Social Care Alliance Scotland. Study materials were provided in a range of accessible formats: digital, paper, as well as video with British Sign Language (BSL) interpretation, subtitles (closed captions) and audio-narration. At recruitment, participants were asked to provide informed signed consent and then to complete a data capture form (Supplemental File 1) to provide information regarding their sensory impairment, medicine regimen*, and use of communication and assistive technologies (*The participants provided a list of medicines using the data capture form and this information was then reviewed and checked with a researcher during a telephone/Zoom call solely for this purpose.). They were also asked to indicate their preferred modes of communication, data collection and sharing that is, provision of data to the research team.

### Data Collection

**Figure 2.**
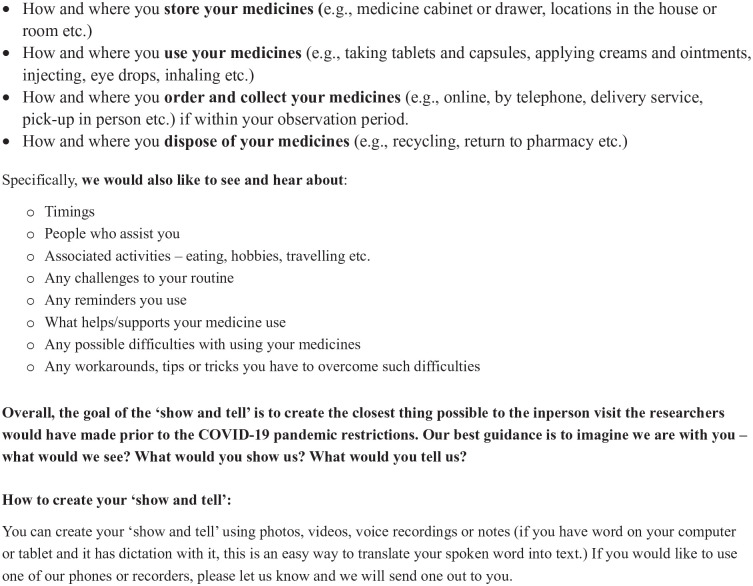
“Show and tell” protocol.

Participants were asked to record episodes of their medicine journey during a 2-week “observation” period using both their own devices for example, Smartphones, cameras or notebooks, or a recorder provided by the research team and took/provided notes. A “Show and Tell” observation protocol (Figure 2) was developed by the research team to record “observations” about each participant’s medicine journey (Faisal et al., 2022).

**Figure 3.**
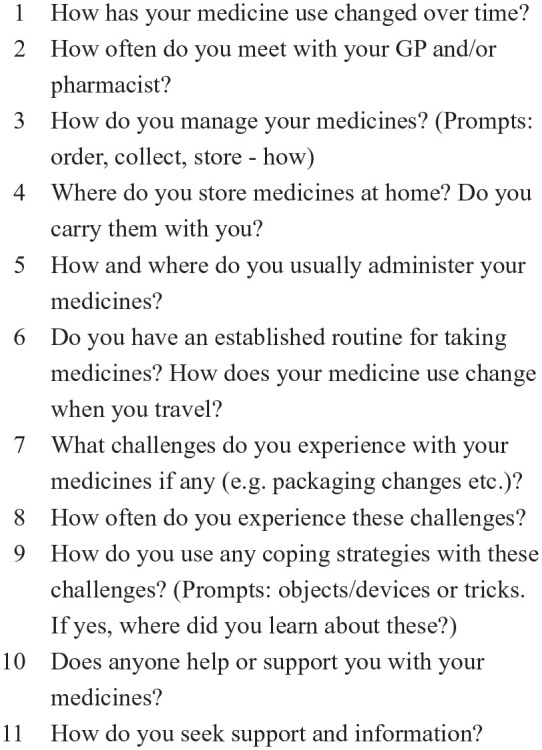
Interview topic guide.

The protocol reflected the five stages of the medicine journey and included information about medicine timing and associated activities for example, mealtimes, people assisting the participant, challenges with medicine use, as well as reminders, workarounds, and the use of technologies or tools to address difficulties and/or support medicine use. Each participant received technical support from the researchers (PF/KB) to record and share their data using their preferred method. Most participants shared their data using their personal email accounts and/or by uploading it to a secure shared internet server. Some participants sent their data by post. Their audio- and video-recordings were discussed with the researchers to gather additional information and clarify existing data, as well as gain a deeper understanding of the context and complexities of each participant’s individual medicine journey. Alongside these discussions, the researchers generated “field notes” to complement the recordings. Following the “observation” period, semi-structured interviews, informed by a topic guide (Figure 3), were completed with each participant, either online using the Zoom platform, or by telephone. The topic guide was informed by the literature and developed and reviewed by the multi-disciplinary research team. The purpose of the interviews was to explore each participant’s medicine journey in greater detail including, routines, challenges, coping strategies, changes over time, and their experiences of engaging with healthcare professionals. The data collection process was piloted with two individuals and as no changes were required that is, none of these data collection tools or processes required to be changed as a result of piloting, the pilot participants’ data were included in the final dataset.

### Data Management and Analysis

Audio-recorded observations and interviews were transcribed by the University of Strathclyde transcription service into intelligent verbatim transcriptions. All transcripts were checked alongside the original recordings for accuracy. The analysis used a combination of inductive and deductive approaches (Ritchie & Lewis, 2013), with the latter reflecting the stages of the medicine journey. These data were coded using an open coding approach informed by the constant comparative method (Strauss & Corbin, 1994). The constant comparative method was initially developed in the context of Grounded Theory and was applied in this study as an inductive approach to reconstruct and reflect the ways older people with sensory impairment manage their medicines at home using their own words.

An iterative approach was used during familiarization with these data and open coding. Three transcripts were initially manually coded and analyzed independently by two researchers [PF/KB] using the constant comparative method. Following coding of the first seven transcripts, an initial codebook was compiled, collated and confirmed, with codes organized into higher-level categories by mapping their association and logical connections. The codebook was used and iteratively refined for analyzing the remaining seven interviews. The researchers reviewed the complete dataset, including interview transcripts, photographs, video- and audio-recordings, participant diary notes and the researcher field notes. The same codebook was used for coding all forms of data for example, photos, diary notes. Differences in codes were continually discussed and compared.

The study received ethical review and approval from the University Ethics Committee (reference: UEC20/72).

## Results

Fourteen individuals participated of whom seven had dual impairment, four had hearing impairment and three had visual impairment (Table 1). Three participants (R11, R12, R19) had been living with their sensory impairment since childhood and the remainder has acquired their sensory impairment(s) later in life.

**Table 1.** Participant Demographic Characteristics (*n* = 14).

Study Id number	Route of recruitment	Sex	Age (years)	Type of sensory impairment	Lives alone	Previous healthcare employment/role	High risk medicine	No. medicinal products	Assistive technologies used
P1	PAG	W	80	Visual	No	No	Digoxin	5	None
P2	PAG	W	67	Hearing	Yes	No	Methotrexate	6	Comfort Contego T800 transmitter (Hearing aid with loop adaptor)
R1	Alliance	W	65	Hearing	No	No	No	6	None
R2	Deafblind Scotland	W	89	Dual	No	Yes (general practice—administration and dispenser role)	No	12	Large print. Magnifiers.
R3	Alliance	M	80	Visual	No	No	Oral hypoglycemic agent.	6 (two of which are combination formulations)	Weekly medicine multi-compartment compliance device (self-filled)
									Magnifying glass. Large font (2—25 size). Seeing aid application on iPhone.
									White cane.
R4	Deafblind Scotland	W	74	Dual	Yes	Yes (general practice—administration role)	Insulin & oral hypoglycemic agents	14 + 5 items for blood glucose testing/hypoglycemic episode management	Loupe magnifier. Magnifying glasses. Lip reads. Large print.
R5	Deafblind Scotland	M	83	Dual	No	No	No	7	Large print, magnifier with light, Ruby 7 magnifier with colors.
R6	Deafblind Scotland	W	66	Dual	Yes	Nurse	Non-steroidal anti-inflammatory drug	12	Mobile phone – text, calls, voice messages; iPAD.
R7	Deafblind Scotland	W	69	Dual	Yes	Nurse	Oral hypoglycemic agent. Azathioprine.	10	Hearing aid. Dosette Box. Rollator (mobility device)
R11	Alliance	W	71	Visual	Yes	Yes (healthcare administration)	Multiple analgesics including codeine, pregabalin, cyclizine.	22	Laptop with maximum magnification.
R12	Alliance	M	78	Dual	No	Yes (military-related training—not a registered health care professional)	Analgesics including dihydrocodeine, gabapentin.	10 (reports not using one analgesic)	Dosette box.
									Magnifying glasses.
									Mobility devices.
									Optomec changes colors, font, computer—laptop, printer for enlarging, audio-descriptions for TV and storytelling, zoom, Teams. Previously used a talking clock.
R14	Personal contact of research team member	M	82	Hearing	No	No	Analgesics e.g. co-codamol, amitriptyline.	10 (but reports not using two products)	None.
R18	Alliance	W	79	Hearing	No	Nurse	No	16	Hearing aids. Previously used assistive system to watch television—doesn’t work any more.
R19	Alliance	M	65	Dual	No	No	Analgesics e.g. tramadol, gabapentin	17	Dosette box with braille when traveling. Reads braille on medicine. packaging. Screen reader—Jaws, Freedom Scientific for 30 years. Murphy—aid to mobility, iPhone, - built in assistive reader, Bluetooth keyboard with iPhone.

*Note.* Alliance (https://www.alliance-scotland.org.uk/) A national (Scotland) charity intermediary organization for health and social care. Deafblind Scotland (https://www.dbscotland.org.uk/) A national (Scotland) charity for people with dual sensory loss. High risk medicine: a medicine which has a “high risk of causing injury or harm if they are misused or used in error” (Scottish Patient Safety Program, 2024). P1, P2: pilot study participants. Their data were included in the dataset (see Methods). R identified for non-pilot participants. Numbers are not continuous because R8-10, R13, R15-17 did not participate following initial discussion about the study. Reasons for non-participation were that data collection was too burdensome, deterioration in health, and, did not want to use technology for data collection/sharing.

PAG = Project Advisory Group members; W = woman; M = man.

The mean age was 75 (SD 7.7) (range 65–89) years, nine were female, and five lived alone. Five of the 14 administrative areas of NHS Scotland were represented. Several participants had worked in health professional roles or in healthcare settings, thus enhancing their health literacy. Three participants had worked as medical secretaries, three as nurses, and one as a battlefield medic (Table 1). The participants used a mean of 11 (SD 5.0) medicines (range 5–22) in a wide variety of formulations, as well as a range of assistive technologies to support the storage and administration of their medicines (Table 1). Whilst not explored specifically, several participants reported difficulties with mobility and dexterity. No participant reported memory and/or cognitive problems.

Recruitment and data collection were undertaken from March 2021 to April 2022. The dataset comprised 14 interviews (of approximately 8 hr’ total duration), 35 photographs, 29 voice- and video- recordings, and written notes. Two participants received assistance with data collection: one from her adult daughter and another from a paid support worker (not paid by the researchers). A further two participants opted for an “interactive observation” with a researcher during which they used live, online interaction to illustrate where their medicines were stored and/or administered.

The results are presented to reflect the stages of the medicine journey (deductive analysis) followed by categories identified by inductive analysis, that is, “medicine management and adherence” and “medicine safety” identified from these data. Quotes are presented in italics, indented and with the participant’s type of impairment [hearing impairment (HI), visual impairment (VI), or dual impairment (DI)] noted and identification number denoted for example, R1.

### The Medicine Journey

#### Ordering and Obtaining Medicines

Participants used using different systems for ordering prescription medicines including online systems or telephoning their general practice or community pharmacy. Individuals with hearing impairment reported difficulty when their practice restricted ordering to telephone-only.

*I was in the town and said, oh, I’ll just see if I can get an appointment. They said, you’ve got to phone up. I said, well, I’m here. No, you’ve got to phone.’ (R18, HI)*

*When you see someone face-to-face, even if it is on Zoom, you can’t [sic] pick things up wrong, how they’re talking, communicating, but you can’t do that by telephone, you don’t have the same prompts, and they can’t see you. (P2, HI)*

The duration of medicine supply varied, with some participants receiving a 2-month supply whereas others had to re-order on a monthly basis. In addition, some participants reported that their medicine supplies were synchronized which meant that they were supplied with all their medicines at the same time, whilst others received asynchronous medicine re-supply at different times throughout the month, which increased the burden of managing their medicines.

In terms of “collecting” or receiving prescription medicines, several participants reported that their medicines were delivered directly to their home from the pharmacy, whilst for others, either the older people with sensory impairment or their representative for example, spouse, collected them in person from the pharmacy.

#### Storage and Administration

**Photo 1a and b.**
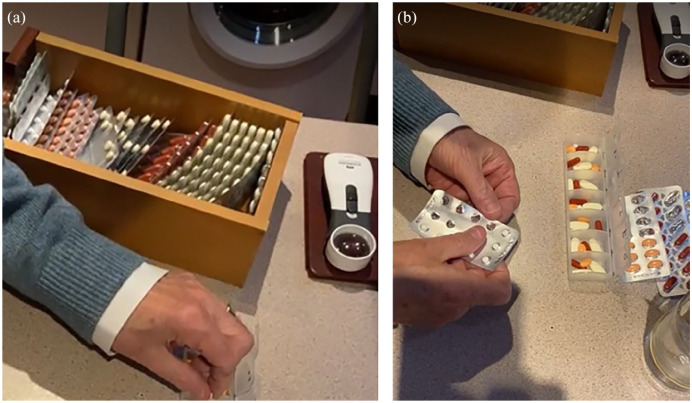
Example of bespoke system used to decant monthly medicine supply into weekly/daily system

The participants used elaborate, bespoke, and complex storage systems and routines for curating their medicines to enable them to remember to administer the right medicine, at the right time and at the right dose. Some kept medicines in their original packaging, while others removed the medicine strips and/or solid dosage forms for example, tablets/capsules, from the strips, and placed them into intermediary containers and locations, which often corresponded to when and what medicine was scheduled to be administered. Examples of bespoke systems included one participant who decanted all their prescription medicines from their original, labeled packs and added them to one large storage container, from which they then decanted weekly doses into their medicine storage device that they filled and used themselves (Photo 1a and b).

Another participant with visual impairment placed medicine packages in a specific order that is, medicines administered in the morning, placed horizontally, medicines administered in the evening placed vertically, to help identify and adhere to his medicines. Two participants who had worked as nurses reported that their clinical experience had informed how they stored and managed their medicines at home.

Several participants spent substantial time every week arranging their medicines into their bespoke systems. These individuals and their systems relied upon consistency in terms of the brand of medicine supplied and its associated appearance in terms of packaging. Changes in packaging and manufacturer were problematic causing participants concern about harmful consequences that could result from misidentification (see Medicines and Safety).

*I order them, they come in from the chemist, I check them, I put them into the cabinet and by checking them, I go, “Hang on, this is different,” and then out comes the magnifying glass. I sometimes go, “What are these?” and “Ah, they are those, they’ve changed the package again.” (R12, DI)*

In addition, unexpected changes to medicine regimen that is, changes that had not been discussed with the older people with sensory impairment and the prescriber or medicine supplier, also impacted personalized systems as well as causing concern and anxiety.

Medicines were typically stored in fixed locations within the home. Most (11) participants stored at least some of their medicines in their kitchen for use first thing in the morning and those that were used last thing at night were typically stored in the bedroom. In all cases, participants administered their medicines in the location in which they were stored.

*Because I’m in the bedroom, in the morning and in the evening that reminds me to do it, yes. The main thing is remembering to take them, but I think I’ve cracked that now. (R1, HI)*

Medicine packaging was problematic for many participants in terms of accessing and removing tablets and capsules prior to administration. This resulted in medicines being dropped and misplaced.

*They’re either difficult to get out but they don’t seem to want to come out, or they come out and they go all over the place. (R12, DI)*

*I’ve got to push very hard and fiddle around and use my nail to pick out the actual pill. At this age I’m sure you know, my hands are not as nimble as they were. (R1, HI)*

#### Disposal

There was minimal discussion of the disposal of medicines by any of the participants.

*It goes into a polythene bag and is taken back to the pharmacist. (R11, VI)*

#### Medicines and Safety

The safe and effective use of medicines relies upon the medicine-user knowing which medicine to use, remembering when to use them, being able to identify them, and the duration for which they should be used.

The participants used different sources of information to support their use of medicines, including the medicine label with the medicine name and dosage instructions, the product information leaflet, and/or pharmacists. The accessibility of information was influenced by the format in which it was provided and the individual’s sensory impairment.

*I had to mention this to my pharmacy, that when they were putting a label on the packaging, not to put it over the braille (R19, DI)*

Whilst some participants used Product Information Leaflets, they frequently reported difficulty with reading the contents.

*I do now read the leaflets, although some of them are quite difficult to read because of the small print. That is quite an issue at times. (P2, HI)*

The extent to which participants sought information and advice from pharmacists varied, with some accessing information routinely:

*Most of the time if I’ve got a query, I will speak to him (the pharmacist). (R11, VI)*

whilst others reported that they tended either not to seek information or when they did, were advised by pharmacy personnel to consult their GP.

*I very rarely speak to the pharmacist at all and certainly not about my regular medication. (R1, HI)*

Some participants experienced uncertainty and ambiguity when using their medicines and a lack of involvement in the decision-making process.

*I take Simvastatin but I don’t have high cholesterol. But it appears to be a rule that for the elderly it extends your lifespan, so therefore you take it, end of story. (R3, VI)*

*I always read those (Patient Information Leaflets) . . .. I shouldn’t be on them for maybe about a year, they should stop. . . . I’ve been on that since..about 2014, 2016. (R14, HI)*

Participants described checking their medicines before administering them and this action prevented one participant (R11) from administering a medicine that had been dispensed to her in error.

*I was given once the wrong dose of [drug name] inhaler. . . . somebody lifted the wrong lot, put them in and .. I got them. . . when I looked .. I thought this is not right. . . (R11, VI)*

Some participants discussed their medicine use and possible side effects with family members, ex-colleagues, and friends. Those living with spouses and other family members, in some cases, relied on their help to collect medicines and discuss medicine use.

Telephone consultations caused concern in terms of medicine safety for participants with hearing impairment.

*When you’re on the telephone you’re having to concentrate. .. it was quite a challenge to concentrate on what she was saying to me as well as taking notes to pass on to the GP. . . My concern is about the mistakes that could be made and the onus that is put on the patient, .. I thought, I hope I’ve got all this right. .. I thought, there could be errors here. (P2, HI)*

One participant discussed being reassured about their medicines because their GP advised them that a medicine review had been done.

*The other thing that reassures me a bit is that I have had one GP at least say to me they have done a medication review on my medication, so ..at least somebody is looking at it and saying can we do this or could we do that? (R11, VI)*

The importance of being able to manage their own medicines was highlighted in terms of maintaining independence and self-esteem.

*I could ask people to do it [fill a weekly Dosette box], but I think sometimes it’s for your own self-esteem .. it can sometimes feel like a small achievement, and I think that is good for your confidence. (P2, HI)*

*I would just be like a baby bird sitting in the nest and someone comes along and stuffs feed in it. .. I would have no say and know nothing, and therefore I wouldn’t even know what I was taking. I think that’s from my own self-esteem point of view and mental well-being, an important thing. (R12, DI)*

### Medicine Management

All participants had fixed routines for their medicine management to facilitate safe and effective medicine use. Several participants used assistive technologies to achieve effective medicine management as well as to address their safety concerns and manage medicine-related risks. Assistive technologies included pill cutters, magnifiers, hearing devices and Smartphone apps, as well as services, such as pre-filled multi-medicine blister* packs prepared by their pharmacy. Multiple compartment devices were the most commonly used types of assistive technology for medicine storage and administration and were used by seven participants. These were either filled by the participants or at the community pharmacy. Two participants used multi-medicine “blister” * packs that were pre-filled and labeled by community pharmacy personnel. (*The term “blister pack” was typically used to describe multi-compartment devices that comprise rigid plastic blisters that correspond to a time of day and day of the week, to assist with medicine adherence.)

## Discussion

The “medicine journey” for older people with sensory impairment is highly variable and reflects both the different degree and type of sensory impairment as well as their abilities, knowledge and skills. A main and novel finding of the study was the substantial burden placed upon participants and the extent to which they had to seek and find their own way of safely managing their medicines. They proactively created relatively fixed routines and bespoke “home-made” medicine safety strategies to manage their medicines. Most of the assistive technologies used were relatively “low-tech” products.

### Medicines Optimization for Older People With Sensory Impairment

When considered through the lens of medicines optimization (Royal Pharmaceutical Society, 2013), the experience of the 14 participants in this study suggests there are areas of clear need for improvement in service delivery, as well as potential gaps in the utilization of assistive technologies. It could be argued that our data suggest that the “patient’s experience” (Principle 1) has not been fully understood based upon the extensive and laborious efforts that many of the participants undertook to ensure their medicines were curated to provide them with confidence about their safe and effective administration.

Whilst the extent to which the participants’ medicine regimen were “evidence-based” (Principle 2) was not assessed directly, the results indicate that there is scope for greater tailoring of regimens to reduce medicine complexity as well as more judicious use of more suitable types of formulations for example, solid dosage forms rather than liquid formulations for people with visual impairment (Wimmer et al., 2017). In so-doing, this could improve medicine adherence and possibly reduce the risk of hospitalization (Wimmer et al., 2017).

From the efforts and examples described by the participants, there is substantial evidence that their “medicine journey” does not reflect Principle 3, “ensure medicines use is as safe as possible” (Royal Pharmaceutical Society, 2013). The burden of managing the safe (and effective) use of medicines appeared to have been placed upon the participants rather than their care providers. Older people are willing to take responsibility for understanding their medicines to enable their safe use (Jallow et al., 2023), however, whilst patient autonomy and responsibility are integral to person-centered care (Ebrahimi et al., 2021; The Health Foundation, 2016; World Health Organisation, 2013), the evidence derived from this study suggests that medicine management can become an excessive and unnecessary burden which could be lightened by care providers at different stages of their journey. Whilst the challenge of medicine burden for older people has been identified previously (Maidment et al., 2020; Morris et al., 2021), this study highlighted the even greater burden associated with the additional challenge of sensory impairment. Research is needed to promote greater attention to prescribing and deprescribing for these patient populations (Rochon et al., 2021). Older people with sensory impairment could benefit substantially from discussion with care providers about how they can manage their medicines and how they could be best supported. Whilst person-centeredness can be operationalized into everyday practice, for example, through the use of consultation tools, more bespoke tools and processes are needed, including ones that address sensory function (Da Costa et al., 2020).

Effective communication and engagement are essential elements of medicine safety (World Health Organisation, 2019b). This includes verbal and written information usually direct to the patient, but sometimes via a proxy, for example, the participant whose spouse collected their medicines because the pharmacy environment prohibited wheelchair access. Older people with sensory impairment are unlikely to achieve optimal safe and effective use of their medicines without substantial changes in the way in which medicine-related information is discussed with and provided to them. The importance of accessible and comprehensible information for all medicine users, irrespective of age and function, to achieve safe and effective administration has been highlighted previously: “If the design and accompanying informational materials of drug products do not match users' needs and capacities, the proper use—hence safety and effectiveness—of even the most efficacious product can be compromised” (Feufel et al., 2020). For older people with sensory impairment, this is of even greater relevance. Whilst not mandatory in the United States (Hayes, 2017), in the European Union (and the UK), all packaging must include the medicine name in braille (Steel, 2013). Although braille users have indicated that this is helpful, many (including participants in this current study) report that medicine labels cover braille when supplied from a pharmacy (Steel, 2013) thereby negating its effectiveness and value.

Principle 4 requires “medicines optimization to be part of routine practice” (Royal Pharmaceutical Society, 2013). From these data gathered and shared by the participants in this study, it would appear that it is older people with sensory impairment who are striving to optimize the aspects of the medicine journey that is within their control. In addition, whilst a range of low technology assistive devices were used by many of the participants, there is considerable scope to explore the use of more advanced technology to manage storage, administration, as well as adherence reminders. The adoption of technologies depends upon users’ skills, abilities and preferences. Previous research into assistive technologies for people with visual impairment reported that whilst participants considered the technology to be potentially useful, they would not use it because it would disrupt their medicine management systems that they had curated over time (Ervasti et al., 2011). As such, a person-centered approach would be essential in identifying and assessing the benefits (and harms), available resources and skills for adopting additional assistive technologies to support rather than disrupt existing strategies.

“Medication Without Harm” is a World Health Organization Global Safety Challenge, for which the “5 Moments for Medication Safety” (World Health Organisation, 2019a) was published, primarily for use by patients but which also requires awareness and input from health and social care professionals. As a generic guide for all medicines and all patients, it does not address many of the medicine journey challenges and patient-derived solutions identified by this current study. It does, however, highlight and endorse the need for greater information sharing and ongoing discussion of medicine-related needs with all patients, and as this study has demonstrated, older people with sensory impairment have additional and predictable requirements that could be better addressed by creating services, products, and strategies that are tailored to their needs and abilities.

### Clinical Implications

The findings have implications for clinicians and clinical services. The provision of more person-centered medicine-related care for older people with sensory impairment is likely to require extended consultations with clinicians to explore and assess the specific needs of individuals. Clinicians are likely to require additional training to assess these needs, to modify regimen to minimize complexity using deprescribing and other strategies, and to suggest or direct patients to assistive technologies to support their medicine journey and reduce overall medicine burden. Few resources exist to promote awareness health and social care professionals of the medicine-related needs of older people with sensory impairment. The findings of this study were used in combination with wider evidence to inform the development of a free, online course to raise awareness (FutureLearn, 2024) but more resources are needed particularly to support consultations prescribing decisions and the assessment of individual patient needs and abilities.

### Strengths and Limitations

Despite the need for remote data collection due to the COVID-19 pandemic, the study produced rich, multifaceted information, contributing to the growing evidence of the potential of remote data collection methods (Humphries et al., 2022). Direct observation of participants would have provided greater insight of the full medicine journey i.e. we were not able to explore the participants’ personal visits to general practices or pharmacies nor their medicine-related consultations with health professionals. These data highlighted the complexities and problems involved with older people with sensory impairment managing their medicines. The intended sample size was 15 participants with five representing each type of sensory impairment. Of the 14 individuals who participated, two who were originally recruited as having visual impairment were subsequently categorized as having dual impairment. The disclosure of additional types of sensory impairment also occurred with an earlier study (Smith et al., 2019). As such, more individuals with dual impairment were included in the sample than was our original intention. The majority of participants were recruited via third sector (charity) organizations and as such, the participants might be more engaged and empowered compared with individuals who are not associated with these organizations. No individuals participated who used BSL, so their specific needs and challenges are not represented in this study. Ideally, had the original sample size been achieved, it would have included a BSL user.

Several participants had worked in healthcare or related employment, and this was often reported as influencing their medicine self-management. It is likely that not all older people will have similar health literacy, skills and cognitive resources to manage their medicines, and that effectively managing their medicines will represent a more significant burden for them.

Future research is needed to explore the needs of older people with sensory impairment during medicine-related consultations with health professionals, including the extent to which prescribers are trained to tailor their consultation and prescribing behavior to reflect the needs of older people with sensory impairment.

## Conclusion

Older people with sensory impairment have diverse and complex medicine needs. Despite robust guidance on medicines optimization, it is clear that older people with sensory impairment could be supported better to reduce their medicine burden by improving current service structures. This study has highlighted specific areas in the medicine journey where person centered approaches to future service development should focus, in particular on communication, environment, and assistive technologies.

## Supplemental Material

sj-docx-1-ggm-10.1177_23337214241253410 – Supplemental material for The Burden of Managing Medicines for Older People With Sensory Impairment: An Ethnographic-Informed Study Supplemental material, sj-docx-1-ggm-10.1177_23337214241253410 for The Burden of Managing Medicines for Older People With Sensory Impairment: An Ethnographic-Informed Study by Peter Fuzesi, Kirsten Broadfoot, Marilyn Lennon, Sabrina Anne Jacob, Leah Macaden, Annetta Smith, Tomas Welsh and Margaret C. Watson in Gerontology and Geriatric Medicine
