# Supplementary material for: The Burden of Managing Medicines for Older People With Sensory Impairment: An Ethnographic-Informed Study
Source: Gerontol Geriatr Med. 2024 May 16;10:23337214241253410. doi: 10.1177/23337214241253410 (PMC11100380; doi:10.1177/23337214241253410)
Supplement: sj-docx-1-ggm-10.1177_23337214241253410 – Supplemental material for The Burden of Managing Medicines for Older People With Sensory Impairment: An Ethnographic-Informed Study [file sj-docx-1-ggm-10.1177_23337214241253410.docx]

**Supplementary File 1**

**Data Collection From**

Our aim is to improve the safe effective use of medicines for older people with sensory (hearing and/or vision) loss. For this, first we need to understand how people with hearing and/or vision loss use medicines, the tips, tricks and strategies they apply and the challenges they face.

The research involves three main parts: 1) an observation, 2) an interview with the participant and 3) further interviews with people who are involved in supporting your medicine use e.g., GP or pharmacist, family members or carers.

**1. Observation**

Before the COVID pandemic, our plan was to observe people and their medicine use in their home, but this is not possible. So, instead we are asking people to use technology to create an observation/story about their medicine use. These observations would look at three things:

- How you order, collect, store and dispose medicines
- How you use medicines throughout the day – routines, habits etc
- Challenges you experience with your medicines
- Who else is involved in your medicine use and how?

**2. Interview**

Once completed, the observations will be discussed in a one-hour interview with a researcher on the phone or through the internet e.g., through Zoom.

**3. Support Interviews**

We’ll ask you to name all the people involved in your medicine use. This might include health and care professionals (e.g., GP, nurse or pharmacist), family members and others. If you agree, we will ask them to participate in a similar interview where we discuss how they support you in using medicines. We will not share any information that you give us in observations of interviews with anyone. You can withdraw from participating in the research up until three weeks after we interviewed you.

The table below outlines the questions we will ask to gather some more information about you initially for the study. These questions will be discussed in either a phone call or through the internet (e.g. Zoom, skype etc). We provide them to you ahead of time as some of them require detailed answers and you may want to have the information ready to hand.

| **Personal** | | | | | | | |
| --- | --- | --- | --- | --- | --- | --- | --- |
| Participant Reference Number (for research team use) | | | |  | | | |
| Could you tell me about your hearing and vision?  Prompt: Is your sensory loss related to a condition? How long have you been living with sensory loss? | | | |  | | | |
| Do you live alone or with someone else (e.g. partner, spouse, family members, paid support)? | | | |  | | | |
| Are there other people who also support or help you (e.g. informal carers, paid carers, neighbours, friends etc)? | | | |  | | | |
| Have you ever worked in healthcare or received training about medicines? | | | |  | | | |
| GP and Audiology at GRI | | | |  | | | |
| General Practitioner | | | |  | | | |
| Pharmacist | | | |  | | | |
| Person 1 | | | |  | | | |
| Person 2 | | | |  | | | |
| **What medicines do you use?**  Prompt: This includes not only tablets and capsules but liquids, inhalers, injections, creams, ointments, patches, eye-dops, suppositories, pessaries, nebulisers, nasal and ear sprays, or any other form of medicine that you use | | | | | | | |
| Medicine | Medicine Name | Formulation e.g. tables, capsules | Strength e.g. 25 mg | | Dose e.g. 50m (2 x 50mg) | Frequency e.g. number of times used each day/week | Any additional information e.g. timing of administration |
| 1 |  |  |  | |  |  |  |
| 2 |  |  |  | |  |  |  |
| 3 |  |  |  | |  |  |  |
| 4 |  |  |  | |  |  |  |
| 5 |  |  |  | |  |  |  |
| 6 |  |  |  | |  |  |  |
| 7 |  |  |  | |  |  |  |
| 8 |  |  |  | |  |  |  |
| 9 |  |  |  | |  |  |  |
| 10 |  |  |  | |  |  |  |
| **Technology and Communication** | | | | | | | |
| Do you use any assistive technology? | | | |  | | | |
| Do you need any of the following methods to make communication and information sharing easier?   - BSL - lip reading - large print - audio-video recording - Braille - Other (please describe) | | | |  | | | |
| Have you had any training in, or worked with information and communication technology? | | | |  | | | |
| What kinds of technology do you use to communicate with people?  e.g., mobile phone, tablet, email | | | |  | | | |
| Do you have an internet connection at home? | | | |  | | | |
| Do you use a smartphone? | | | |  | | | |
| Do you use email? Can you send attachments? | | | |  | | | |
| Do you use social media? Which platforms? | | | |  | | | |
| Do you have people who can help you when using technology? | | | |  | | | |
| What would be your preferred way of recording your medicine use? | | | |  | | | |
